# Trends of Hospital Admissions Due to Congenital Anomalies in England and Wales between 1999 and 2019: An Ecological Study

**DOI:** 10.3390/ijerph182211808

**Published:** 2021-11-11

**Authors:** Abeer F. R. Alanazi, Abdallah Y. Naser, Prisca Pakan, Atheer F. Alanazi, Alyamama Abdulaziz A. Alanazi, Zahra Khalil Alsairafi, Fatemah M. Alsaleh

**Affiliations:** 1 Department of Pharmaceutical and Biological Sciences, UCL School of Pharmacy, London WC1E 6BT, UK; abeer.alanazi.18@ucl.ac.uk; 2 Department of Applied Pharmaceutical Sciences and Clinical Pharmacy, Faculty of Pharmacy, Isra University, Amman 11622, Jordan; abdallah.naser@iu.edu.jo; 3 Department of Microbiology, Medical Faculty, University of Nusa Cendana, Kupang 09200, Indonesia; priscapakan@staf.undana.ac.id; 4 Department of Biology, Ministry of Health, Riyadh 11176, Saudi Arabia; atheer.fehaid@gmail.com; 5 Department of Biological Sciences, School of Biological and Chemical Sciences, Queen Mary University of London, London E1 4NS, UK; a.alanazi@se20.qmul.ac.uk; 6 Department of Pharmacy Practice, Kuwait University, Kuwait City 12037, Kuwait; zahra.alsairafi@hsc.edu.kw

**Keywords:** England, hospitalisation, congenital, United Kingdom, Wales

## Abstract

*Objectives*: To investigate the trends in congenital anomalies-related hospital admissions in England and Wales. *Methods*: This was an ecological study that was conducted using hospital admission data taken from the Hospital Episode Statistics database in England and the Patient Episode Database for Wales. Congenital malformations, deformations and chromosomal abnormalities hospital admissions data were extracted for the period between April 1999 and March 2019. *Results*: Hospital admission rate increased by 4.9% [from 198.74 (95% CI 197.53–199.94) in 1999 to 208.55 (95% CI 207.39–209.71) in 2019 per 100,000 persons, trend test, *p* < 0.01]. The most common hospital admissions causes were congenital malformations of the circulatory system, the musculoskeletal system, genital organs, and the digestive system. The most notable increase in hospital admissions rate was observed in congenital malformations of the respiratory system (1.01-fold). The age group below 15 years accounted for 75.1% of the total number of hospital admissions. Males contributed to 57.5% of the whole number of hospital admission. Hospital admission rate between females was increased by 6.4% [from 162.63 (95% CI 161.10–164.16) in 1999 to 173.05 (95% CI 171.57–174.54) in 2019 per 100,000 persons]. Hospital admission rate between males was increased by 3.4% [from 236.61 (95% CI 234.72–238.50) in 1999 to 244.70 (95% CI 242.92–246.49) in 2019 per 100,000 persons]. *Conclusions*: Males had a higher percentage of hospitalisation compared to females. Further studies to investigate the factors associated with higher hospitalisation rate among males are needed.

## 1. Introduction

Congenital malformations, deformations and chromosomal abnormalities are also known as congenital anomalies or birth defects [1]. Congenital anomalies (CAs) are defined as behavioural, structural, metabolic, and functional disorders present at childbirth [2]. These congenital disorders can be diagnosed before or after childbirth [1,2]. Many causes of congenital anomalies are not yet well known [1]. Still, the etiologies of congenital anomalies are estimated to be micronutrient deficiencies, single gene defects, multifactorial inheritance, chromosomal disorders, and environmental teratogens [3]. Moreover, low-income may be an indirect reason for congenital anomalies; about 94% of severe congenital anomalies occur in low- and middle-income countries. Related to this, in low- and middle-income countries, pregnant women are more susceptible to malnutrition, reduced access to screening and healthcare, or increased exposure to factors such as alcoholism and infection [1]. Besides, in developing countries, prevalent maternal infectious diseases, like syphilis and rubella, are a cause of congenital anomalies [4]. Old maternal age further raises the risk of chromosomal abnormalities such as Down’s Syndrome [1].

Congenital anomalies are a universal health problem and are one of the principal causes of infant morbidity and death. In high-income countries, about 30% of all mortality in children (<5 years old) is due to congenital anomalies [5]. Annually, an estimated 7.9 million children are born with severe congenital anomalies; the 3.2 million who survive may develop disabilities later in life, and there are 3.3 million deaths among children (<5 years old) associated with congenital anomalies [6]. Besides, an estimated 295,000 newborns die within four weeks of childbirth annually around the world because of congenital anomalies [1]. In eleven European Registration of Congenital Anomalies and Twins (EUROCAT) countries, the mean of congenital anomaly-related infant death was purportedly 1.1 per 1000 births, with more elevated rates where termination of pregnancy for fetal anomaly (TOPFA) is unlawful. The rate of congenital anomalies among stillbirths was 0.6 per 1000, and the mean of TOPFA prevalence was 4.6 per 1000, about three times more common than infant and stillbirths mortalities together [7].

Various prior studies concluded that the proportion of congenital anomalies differed from country to country, with 4.2% in Pakistan, 2.5% in Egypt, 2.0% in Ethiopia, 1.9% in India, and 0.6% in Hong Kong [4,8,9,10,11]. In England, a total of 13,400 children with one or more congenital anomalies was reported to the National Congenital Anomaly and Rare Disease Registration Service (NCARDRS) from 628,171 gross births (stillbirths and live births) in 2018. This presents an overall birth prevalence of 213.3 per 10,000 gross births. That means for every 47 births (stillbirths and live births), there is one case of congenital anomalies [12].

Congenital anomalies constitute 15–30% of all hospital admissions among children; they need care at a relatively higher cost than other hospital admission cases, which significantly affects the community and families [13]. Besides the social, economic, and family impact of the disease, the chronicity of the disease raises the number of hospital admissions and readmissions, increasing the risk of clinical complications and exacerbating the severity of cases [14,15]. This study aims to investigate the trends in congenital anomaly-related hospital admissions in England and Wales between 1999 and 2019.

## 2. Methods

### 2.1. Study Sources and Population

This was an ecological study using publicly available data extracted from the Hospital Episode Statistics (HES) database in England [16] and the Patient Episode Database for Wales (PEDW) for the period between April 1999 and April 2019 [17]. They have been used previously to explore the trends of different health outcomes and the associated hospital admissions [18,19,20,21]. The hospital episode statistics database and patient episode database for Wales record all hospital admissions, outpatients and accident and emergency (A&E) activities performed at all NHS trusts and any independent sector funded by NHS trusts. The hospital episode statistics data can be processed and utilised for secondary objectives not correlated to direct patient care such as health service planning and research.

The HES and PEDW databases contain hospital admission data for patients from all age groups which are subdivided into four categories; below 15 years, 15–59 years, 60–74 years, and 75 years and above. Congenital malformations, deformations and chromosomal abnormalities hospital admissions using the 10th version of the International Statistical Classification of Diseases (ICD) system (Q00–Q99). HES and PEDW data are checked regularly to ensure their validity and accuracy [16,22]. Data collection regarding the main reason of admission done using the Patient Administration System (PAS) at hospitals during patient stay and collected on a monthly basis from hospitals. There is one unique ICD-code for each admission, which is the main reason for hospital admission. Therefore, the extracted and presented data in this study is based on the primary diagnose upon hospital admission. To calculate the yearly hospital admission rate, we collected mid-year population data for the period between 1999 and 2019 from the Office for National Statistics (ONS) [23].

### 2.2. Statistical Analysis

Hospital admission rates with 95% confidence intervals (CIs) were calculated using the finished consultant episodes of hospital admission divided by the mid-year population. Annual hospital admissions rates for male were calculated using the number of hospital admissions related to each type of congenital anomalies for each age group among males divided by the mid-year population for males of the same age group of the same year. Similar procedure was followed to calculate annual hospital admissions rates for females. We used the chi-squared test to assess the difference between the hospital admission rates. The trend in hospital admissions was assessed using a Poisson model. All analyses were conducted using SPSS version 25 (IBM Corp, Armonk, NY, USA).

## 3. Results

The total yearly number of hospital admissions for diverse causes increased by 19.6% from 103,621 in 1999 to 123,962 in 2019, expressing an increase in hospital admission rate of 4.9% [from 198.74 (95% CI 197.53–199.94) in 1999 to 208.55 (95% CI 207.39–209.71) in 2019 per 100,000 persons, trend test, *p* < 0.01].

The most common hospital admissions causes were congenital malformations of the circulatory system, the musculoskeletal system, genital organs, the digestive system, and other congenital malformations which accounted for 20.1%, 19.5%, 14.2%, 12.0%, and 10.1%, respectively (Table 1).

**Figure 1:**
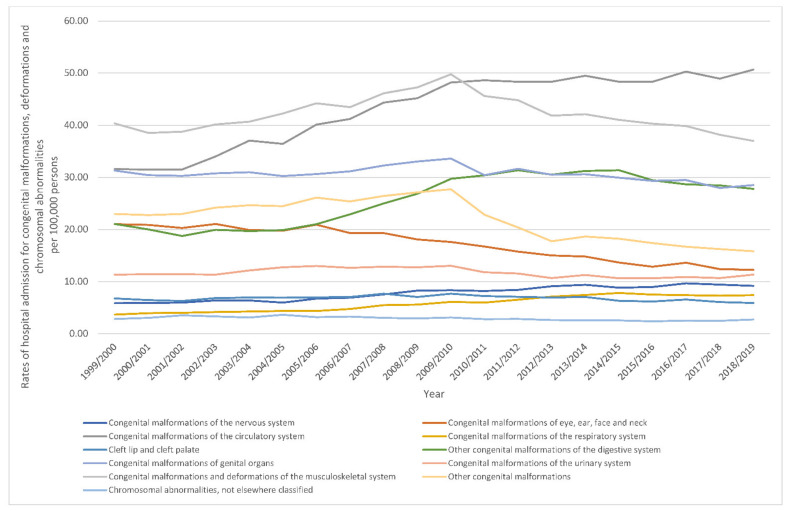
Rates of hospital admission for congenital malformations, deformations and chromosomal abnormalities in England and Wales stratified by type between 1999 and 2019.

The most notable increase in hospital admissions rate was observed in congenital malformations of the respiratory system with 1.01-fold. Furthermore, hospital admissions rate for congenital malformations of the circulatory system, the nervous system, and the digestive system were increased by 60.5%, 56.6%, and 32.0%, respectively. Still, congenital malformations, deformations and chromosomal abnormalities hospital admissions rate for congenital malformations of eye, ear, face and neck, other congenital malformations, cleft lip and cleft palate, genital organs, the musculoskeletal system, chromosomal abnormalities not elsewhere classified, and the urinary system were decreased by 41.7%, 31.2%, 12.8%, 8.8%, 8.3%, 3.8%, and 0.2, respectively (Table 2, Figure 1).

**Figure 2:**
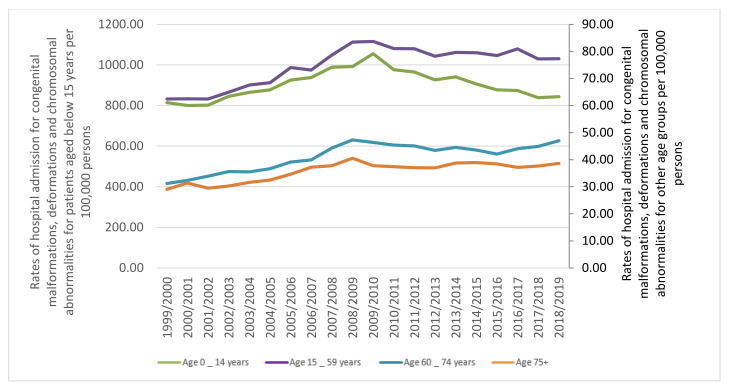
Rates of hospital admission for congenital malformations, deformations and chromosomal abnormalities in England and Wales stratified by age group.

The age group below 15 years accounted for 75.1% of the total number of hospital admissions, followed by the age group 15–59 years with 20.8%, the age group 60–74 years with 2.8%, and then the age group 75 years and above with 1.3%. Rates of hospital admission among patients aged below 15 years increased by 3.7% [from 814.10 (95% CI 808.51–819.70) in 1999 to 843.89 (95% CI 838.42–849.37) in 2019 per 100,000 persons]. Rates of hospital admission among patients aged 15–59 years increased by 23.8% [from 62.44 (95% CI 61.57–63.32) in 1999 to 77.29 (95% CI 76.36–78.21) in 2019 per 100,000 persons]. Rates of hospital admission among patients aged 60–74 years increased by 50.5% [from 31.23 (95% CI 29.91–32.54) in 1999 to 46.99 (95% CI 45.59–48.39) in 2019 per 100,000 persons]. Rates of hospital admission among patients aged 75 years and above increased by 32.9% [from 29.09 (95% CI 27.41–30.78) in 1999 to 38.65 (95% CI 36.94–40.36) in 2019 per 100,000 persons] (Figure 2).

**Figure 3:**
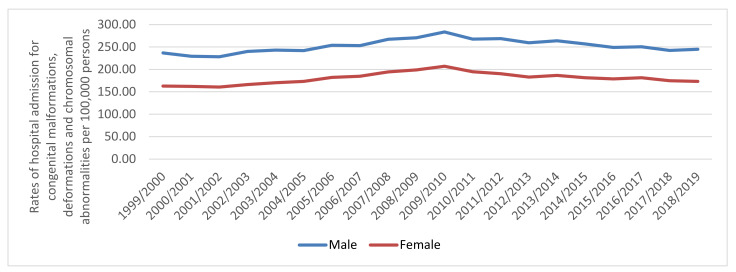
Rates of hospital admission for congenital malformations, deformations and chromosomal abnormalities in England and Wales stratified by sex.

A whole of 2,395,135 hospital admission episodes were recorded in England and Wales during the study time. Males contributed to 57.5% of the whole number of hospital admission accounting for 1,378,138 episodes by a mean of 68,906 per year. Hospital admission rate between females was increased by 6.4% [from 162.63 (95% CI 161.10–164.16) in 1999 to 173.05 (95% CI 171.57–174.54) in 2019 per 100,000 persons]. Hospital admission rate between males was increased by 3.4% [from 236.61 (95% CI 234.72–238.50) in 1999 to 244.70 (95% CI 242.92–246.49) in 2019 per 100,000 persons] (Figure 3).

### 3.1. Admission Rate by Sex

**Figure 4:**
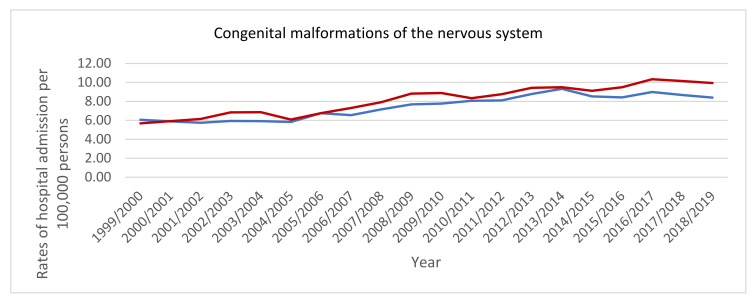

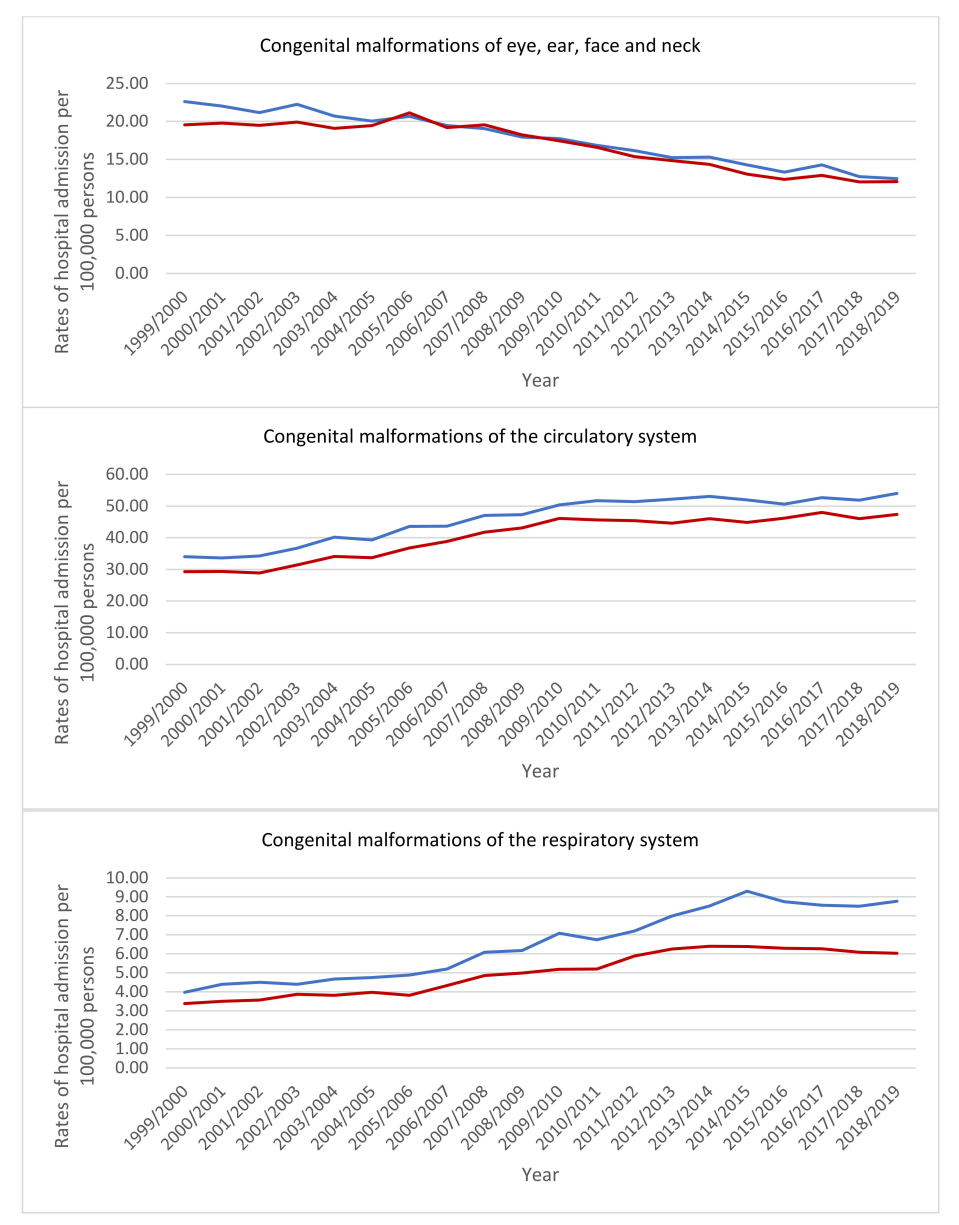

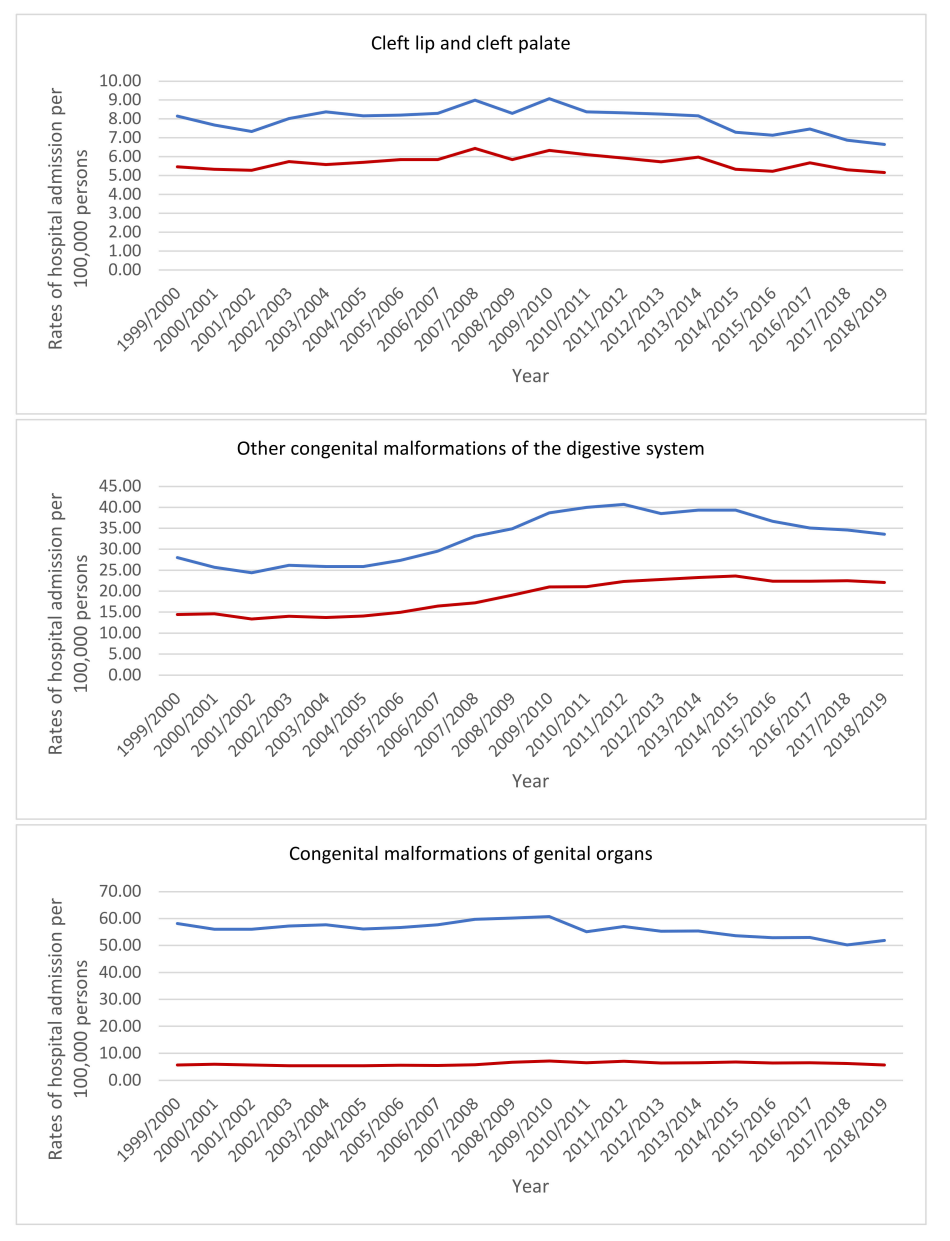

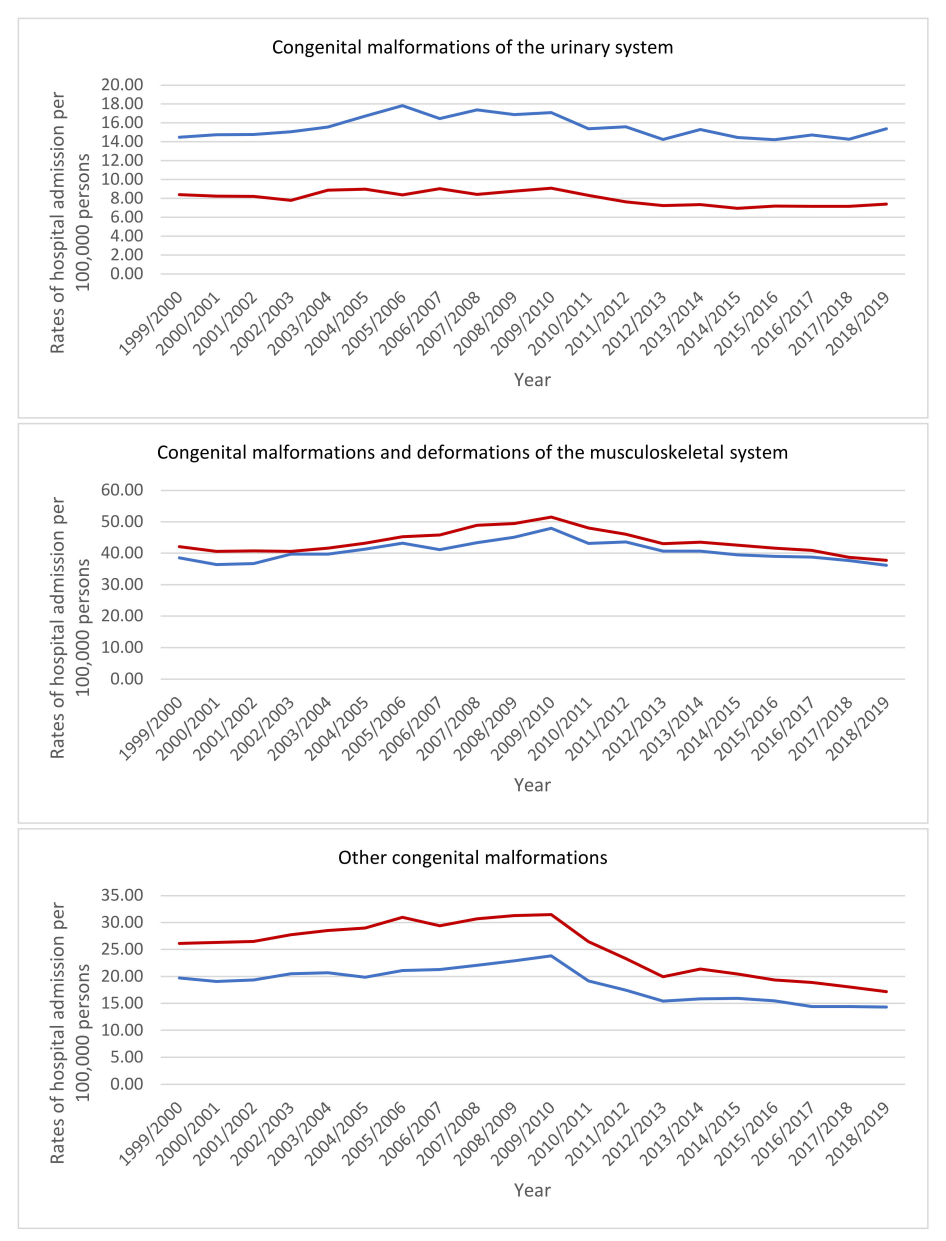

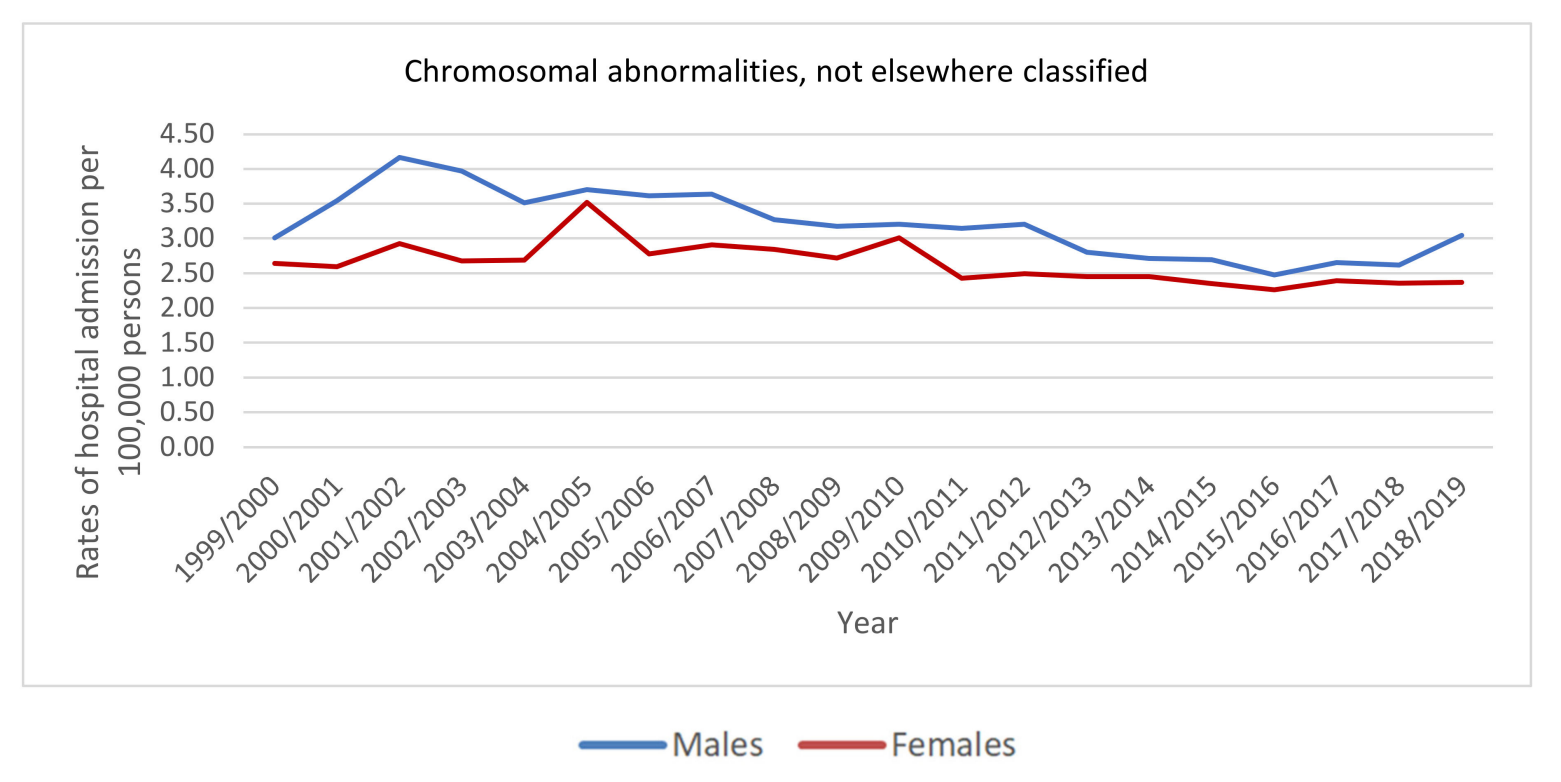
Hospital admission rates for congenital malformations, deformations and chromosomal abnormalities in England and Wales stratified by sex.

Hospital admission rates for congenital malformations of eye, ear, face and neck, the circulatory system, the respiratory system, cleft lip and cleft palate, the digestive system, genital organs, the urinary system, and chromosomal abnormalities, not elsewhere classified were higher among males compared to females, while hospital admission rates for congenital malformations of the nervous system, the musculoskeletal system, and other congenital malformations were higher among females compared to males (*p* < 0.05) (Figure 4).

### 3.2. Admission Rate by Age Group

**Figure 5:**
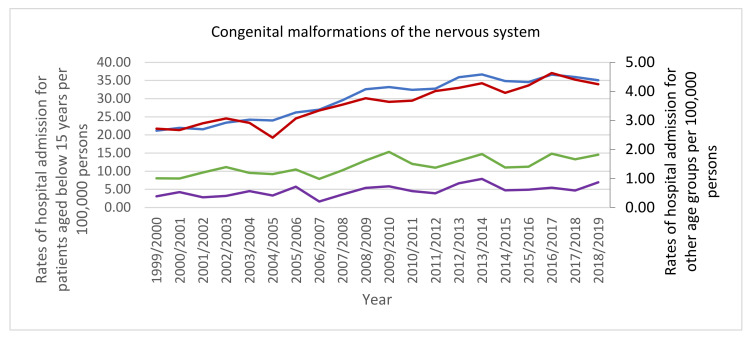

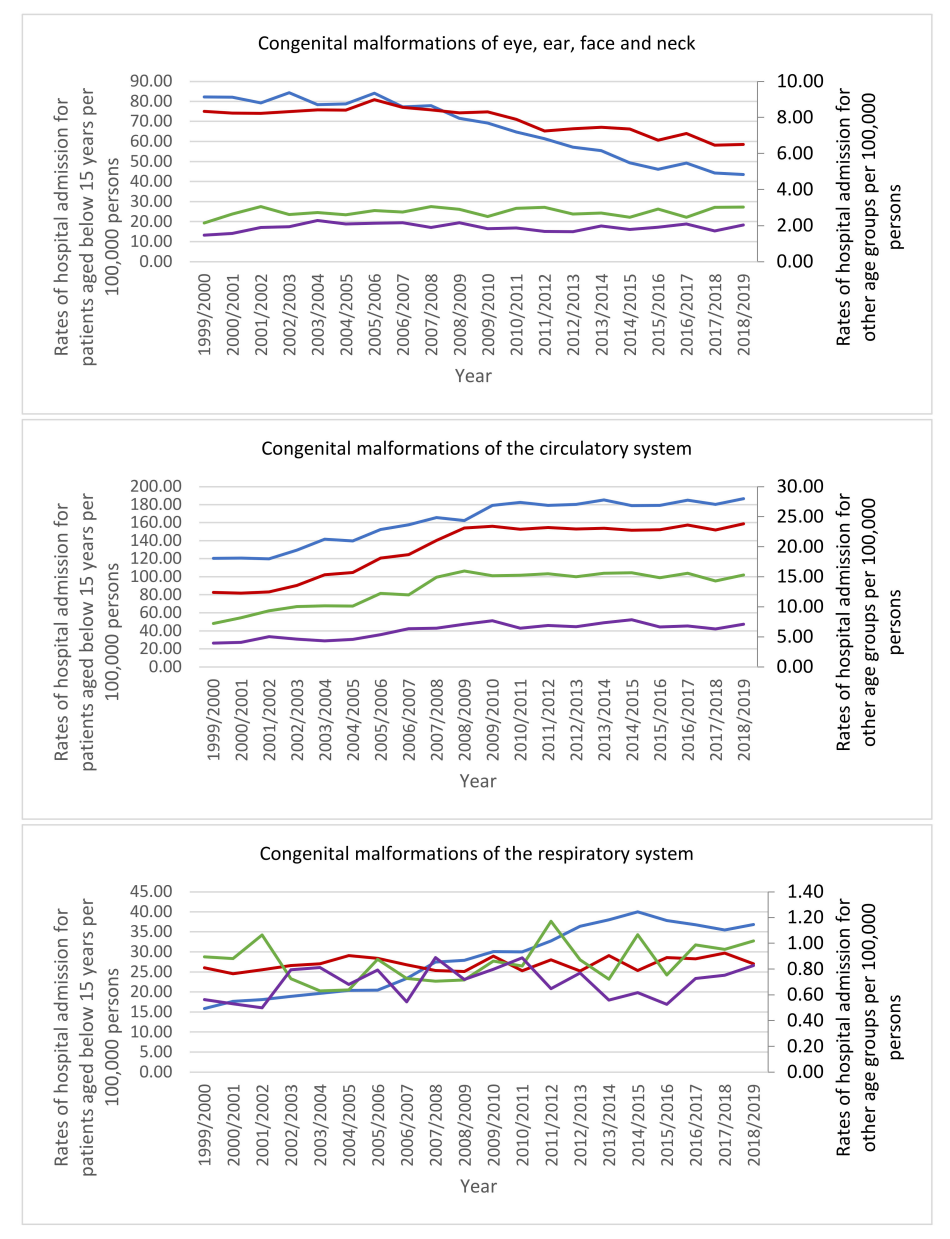

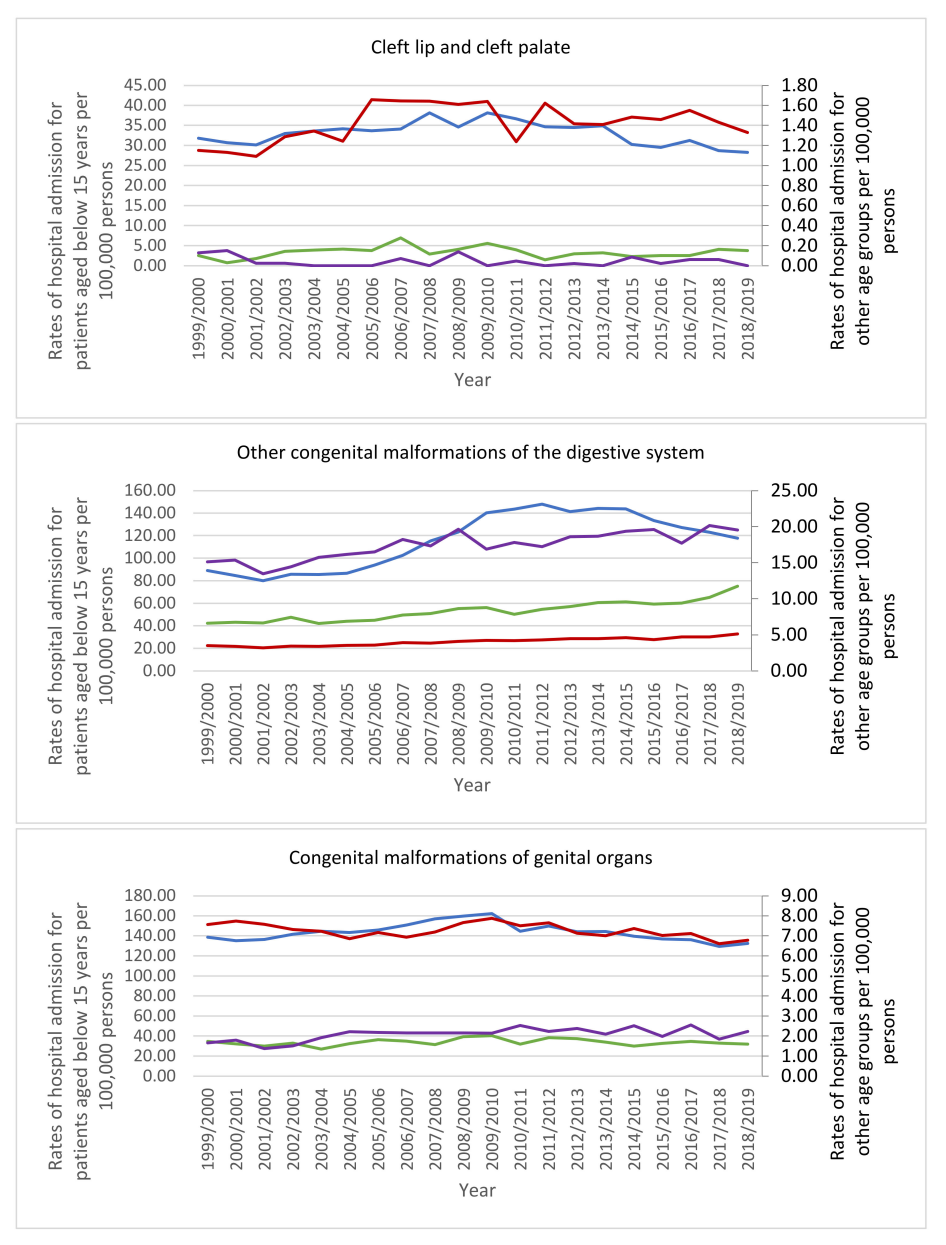

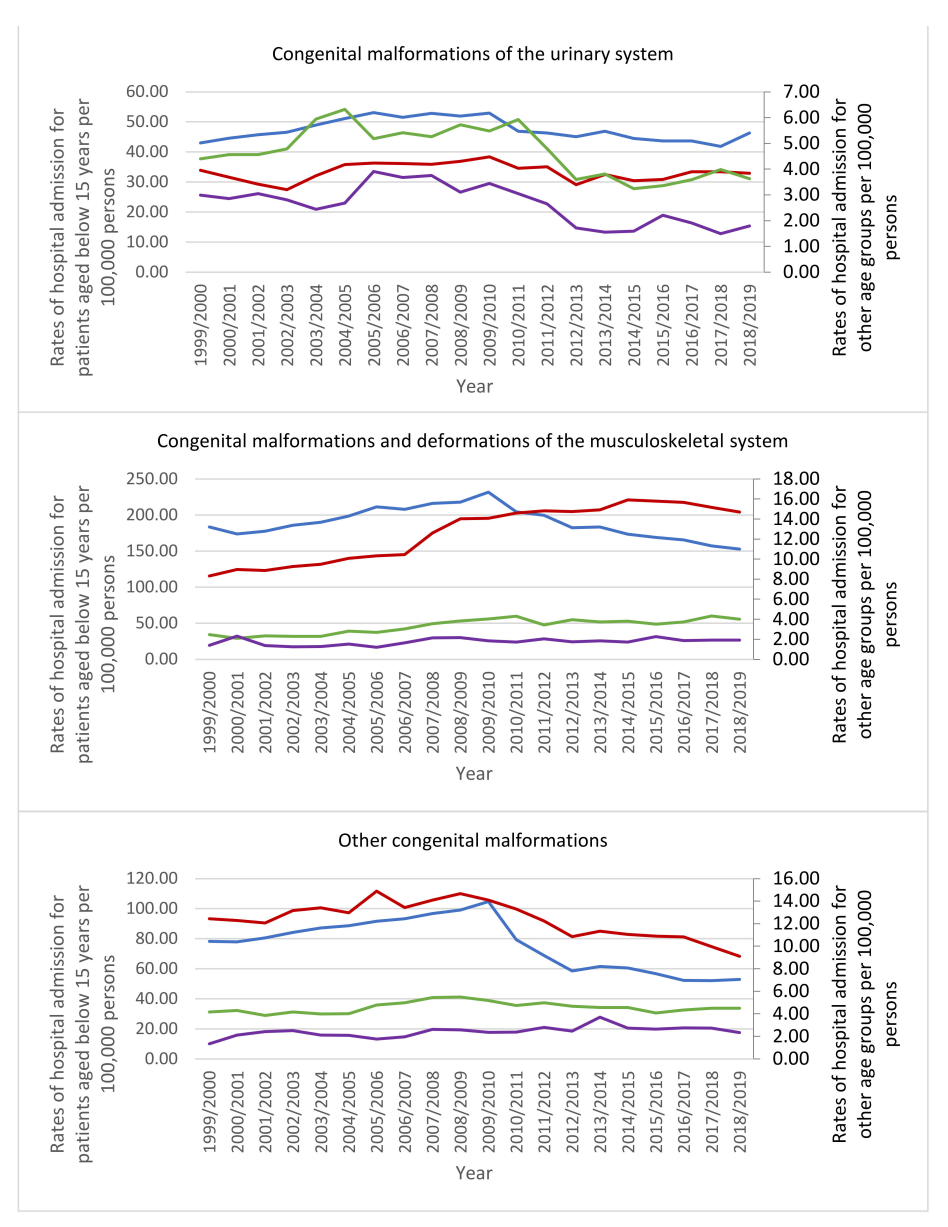

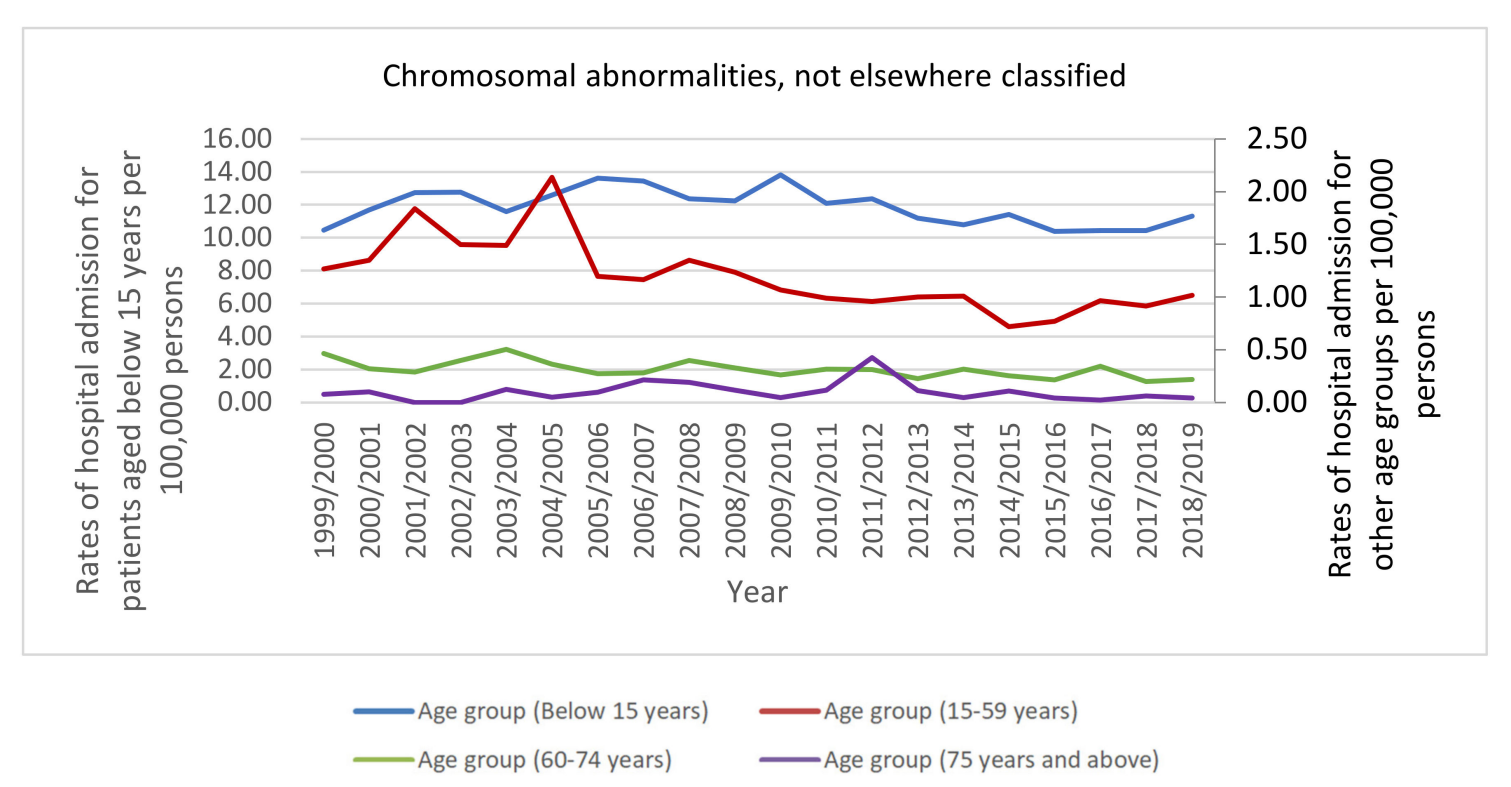
Hospital admission rates for congenital malformations, deformations and chromosomal abnormalities in England and Wales stratified by age group.

The bulk of hospital admissions were observed to be inversely related to age (more common among the age group below 15 years, Figure 5). That includes the following: congenital malformations of the nervous system, eye, ear, face and neck, the circulatory system, cleft lip and cleft palate, the musculoskeletal system, other congenital malformations, and chromosomal abnormalities, not elsewhere classified. Besides, hospital admissions due to congenital malformations of genital organs were more common among the age group: below 15 years, 15–59 years, 75 years and above, and 60–74 years, respectively. Hospital admissions due to congenital malformations of the respiratory system and the urinary system were more common among the age group: below 15 years, 60–74 years, 15–59 years, and 75 years and above, respectively. Hospital admissions due to other congenital malformations of the digestive system were more common among the age group: below 15 years, 75 years and above, 60–74 years, and 15–59 years, respectively.

### 3.3. Admission Rate Excluding Gender-Specific Congenital Anomalies

The total yearly number for hospital admissions due to congenital anomalies excluding those who are gender-specific increased by 22.5% from 86,889 in 1999 to 106,460 in 2019, expressing an increase in hospital admission rate of 7.5% [from 166.65 (95% CI 165.54–167.75) in 1999 to 179.11 (95% CI 178.03–180.18) in 2019 per 100,000 persons, trend test, *p* < 0.01].

**Figure 6:**
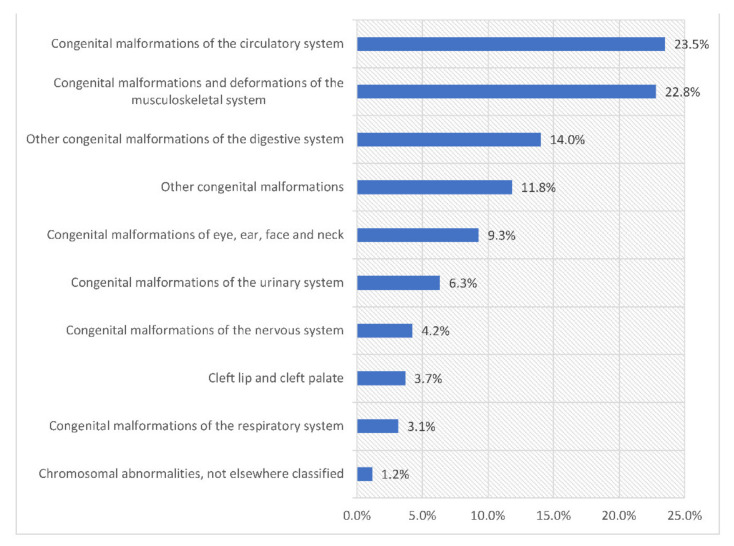
Percentage of congenital malformations, deformations and chromosomal abnormalities hospital admission from total number of admissions.

The most common hospital admissions causes were congenital malformations of the circulatory system, congenital malformations and deformations of the musculoskeletal system, other congenital malformations of the digestive system, and other congenital malformations which accounted for 23.5%, 22.8%, 14.0%, and 11.8%, respectively (Figure 6).

**Figure 7:**
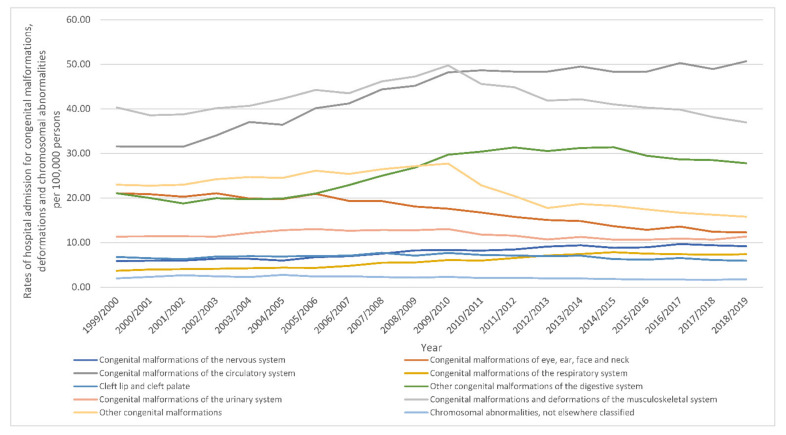
Rates of hospital admission for congenital malformations, deformations and chromosomal abnormalities in England and Wales stratified by type between 1999 and 2019 excluding gender-specific anomalies.

During the past two decades, the most notable increase in congenital malformations, deformations and chromosomal abnormalities hospital admissions rate was observed in congenital malformations of the respiratory system with 1.01-fold. Furthermore, congenital malformations, deformations and chromosomal abnormalities hospital admissions rate for congenital malformations of the circulatory system, congenital malformations of the nervous system, and other congenital malformations of the digestive system were increased by 60.5%, 56.6%, and 32.0%, respectively. Still, congenital malformations, deformations and chromosomal abnormalities hospital admissions rate for congenital malformations of eye, ear, face and neck, other congenital malformations, cleft lip and cleft palate, chromosomal abnormalities, not elsewhere classified, congenital malformations and deformations of the musculoskeletal system, and congenital malformations of the urinary system were decreased by 41.7%, 31.2%, 12.8%, 10.6%, 8.3%, and 0.2, respectively (Figure 7).

## 4. Discussion

The total yearly number of hospital admissions increased by 19.6% from 103,621 in 1999 to 123,962 in 2019. In 2018 in the UK, the National Congenital Anomaly and Rare Disease Registration Service (NCARDRS) report showed that 9836 live births have been diagnosed with a congenital anomaly; of those, 699 died in infancy, giving an infant mortality rate of between 10.4 and 12.0 per 10,000 live births [12]. In addition, according to the NCARDRS report, there were around 13,400 babies with one or more congenital anomalies in 2018 [24]. Observational data on the incidence of hospital admissions due to congenital diseases in the UK are limited, and we were unable to compare the results with previous studies. However, some other studies worldwide also reported an increase in the overall trend of congenital anomalies. A retrospective study of hospitalisation (2005 to 2015) due to congenital anomalies in Australia reported an overall increase in the trend of hospitalisation among patients with congenital anomalies, which was similar to our results [25]. This study reported that male to female hospital admission ratio was 1.5:1. There was an increase in trend of admissions over the study period. Additionally, the most common causes of hospital admission related to congenital anomalies involved the cardiovascular system and digestive system anomalies [25].

During the study period between 1999 and 2019, the rate of hospital admissions increased for specific types of congenital anomalies and stabilised or decreased for others. Starting from the year 2009/2010, we noticed interested trends of hospital admissions for specific types of congenital anomalies. This could be related to improvement in the diagnostic ability related to these specific abnormalities, which facilitate pregnancy termination. The Department of Health and Social Care in England and Wales reported that the overall age-adjusted abortion rate started to decrease in 2010 until 2016 and returned to increase afterward. However, during the same period the abortion rate for women aged over 35 years increased, which itself could contribute to the decrease in specific types congenital anomalies related to this maternal age. While in the same period, the abortion rate for women aged under 18 years decreased, which could lead to an increase in specific types congenital anomalies related to this younger maternal age group [26]. The NCARDRS report revealed that for women aged between 30 and 34 years at delivery, the birth prevalence of all congenital anomalies was insignificantly lower than in those aged between 25 and 29 years, presenting 187.1 and 192.2 per 10,000 total births, respectively. In contrast, in mothers aged 35 to 39 years, the birth prevalence was significantly higher (229.9 per 10,000 total births) [12].

For mothers under 20 years, rates of congenital anomalies were significantly similar to those aged 20 to 24 years but significantly higher than in those aged between 30 and 34 years. There is a well-established association between higher maternal age and specific genetic anomalies, such as Down’s syndrome, particularly in women aged over 40 years at delivery. Women over 40 years of age had a seven times higher rate of genetic disorders compared to those under 20. Other congenital anomalies, such as gastroschisis, which is an abdominal wall anomaly, are more common among women aged under 20 years. The prevalence of gastroschisis is 19.9 and 0.4 per 10,000 births in women aged under 20 and over 40 years, respectively [12].

The increase in the trend of hospitalisation due to congenital diseases can be related to multiple reasons which are also risk factors for the development of congenital anomalies. The management of congenital malformations has improved, leading to less mortality among children. This means more surviving children who need further therapy and hospitalizations. For example, the continuous development of interventions for the management of congenital heart diseases (CHD) has had a significant impact in improving the survival rate of affected patients [27].

Congenital anomalies may be the result of one or combination of factors such as socioeconomic (low-income) and demographic, genetic (gene mutations), maternal infections (such as syphilis and rubella), maternal nutritional status (such as folate insufficiency) or environment, however, it is often difficult to identify the exact causes [1]. Other factors may include consanguineous, which has been reported to as an important and growing risk factor in the UK with the growing percentages of some immigrant populations such as Pakistani origins [28,29]. Other risk factors include advance maternal age and full term pregnancies [30]. Besides, previous studies reported that antihypertensive and antiepileptic therapies are associated with different types of CVS congenital anomalies [31,32]. Shawky et al. reported that there is an association between early antepartum hemorrhage during pregnancy and congenital anomalies [33]. Other risk factors that were identified in a previous study in Egypt to be linked with having babies with congenital anomalies are old-aged parents, exposure to chemicals and pesticides during pregnancy, receiving non-prescribed medications and excessive vitamin A during pregnancy, and living near mobile strengthening stations [34].

In our study, we found that congenital heart defects were the most common and accounted for the highest percentage across the study period. These results were also consistent with previous studies [1]. In a study by Dolk et al., the authors reported that congenital heart defects (CHD) were the most common non-chromosomal subgroup, at 6.5 per 1000 births, followed by limb defects (3.8 per 1000), anomalies of the urinary system (3.1 per 1000), and nervous system defects (2.3 per 1000) [35]. Similarly, in another study that was conducted in the UK, the authors reported that congenital heart diseases had the highest prevalence (50 per 10,000 births), followed by anomalies of the limbs (49 per 10,000 births), and digestive system anomalies (47 per 10,000), respectively [36], with other studies worldwide also reporting similar results [34,37]. The authors of this study also reported that the prevalence of congenital anomalies due to other systems was declining, including those of the ear (−88%), congenital heart disease (−69%), anomalies of integument (−67%), nervous system anomalies (−61%), anomalies of the limbs (−54%), and urogenital (including renal) anomalies (−31%). This was also noticed in our study as congenital anomalies due to cleft palate, cleft lip, urinary system and genital system were stable across the study period. However, it is also important to note that these results pertained to the prevalence in the general population, while our study was specific to hospital admissions.

We found that most of the congenital admissions were higher among male patients. Multiple studies have confirmed that the prevalence of congenital anomalies is higher among males compared to females [34,38,39]. Our results were similar to those of a previous study in Brazil, in which the authors reported that around 66.9% of total cases were male patients. In another study in the UK (1985–2003), the authors investigate the sex distribution in various congenital anomalies. The authors reported that male fetuses were significantly more prevalent in pregnancies affected by a congenital anomaly than female fetuses (RR, male vs. female = 1.15; 95% CI: 1.11–1.19), but there was significant heterogeneity between subtypes (*p* < 0.001) [38]. Also, similar results were reported in Morocco, where the authors investigated sex differences in the prevalence of congenital anomalies. The authors reported that there was a predominant male ratio (57%) among the study population. Although these results pertained to the general population and pregnant women, they reflect the actual prevalence and real-world data; thus, they are likely to apply to the hospital admission rate as well [40]. Although, our results were consistent with the literature, there have remain an uncertainty of a physiological, biological, or endocrinological causes to explain these differences between males and females [39]. In addition, we found that patients aged under 15 years old had a higher percentage of hospital admissions compared to those older than 15 years. These results were also consistent with previous studies in the literature as they suggest that death and morbidity due to congenital anomalies are likely to be in the very early stages of life. The increase in the rate of hospital admissions for patients aged under 15 years can be attributed to chromosomal disorders and early surgical treatment for correctable congenital malformations. This can be the cause of the lesser rate of hospitalization among the older age group (above 15 years) since patients with surgically treated malformations may need only follow-ups in outpatient clinics for years after the treatment. In contrast, those with chromosomal anomalies may be admitted many times at any age due to problems related to the malformation itself, vulnerability to infections, or other associated illnesses.

Several measures/interventions have been introduced worldwide to reduce the number of congenital anomalies, including vaccination programmes (especially against the rubella virus for children and women), enhancing knowledge and awareness of the importance of a healthy diet and healthy weight during pregnancy, promoting adequate intake of folic acid or iodine during pregnancy, and avoiding harmful substances such as tobacco, hazardous substances, medications and radiation during pregnancy [1]. The WHO recommended fortification of staple foods or supplementation, and adequate antenatal care to decrease the burden of congenital anomalies [1]. Additionally, the WHO recommended the following interventions to decrease the frequency of certain congenital anomalies: (1) ensuring healthy diet for girls and mothers (including variety of vegetables, fruits and adequate dietary intake of vitamins and minerals), (2) controlling diabetes mellitus prior to and during pregnancy, (3) strengthening healthcare professionals’ knowledge and education in promoting prevention of congenital anomalies, and (4) screening for infections [1].

Preconception screening, peri-conception screening and neonatal screening including basic reproductive health practices, as well as medical genetic screening are vital reproductive healthcare services that play a key role in the early detection of congenital anomalies [1,41]. Early detections and screening programs including ultrasonography have improved in the last years. In the UK, a mid-pregnancy or anomaly scan is usually carried at the 20th week of pregnancy, which leads to a late diagnosis of some fetal malformations, such as anencephaly, omphalocele, and limb anomalies that can be detected earlier at 10–14 weeks of pregnancy [42,43,44]. Better diagnostics procedures (such as Magnetic Resonance Imaging (MRI), genetic testing, Molecular testing of the chorionic villus sample (CVS) or amniocytes, fetal blood sample, a direct biopsy of fetal tissue, and Non-invasive prenatal diagnosis (NIPD)) help in the early detection of congenital anomalies during the perinatal period (earlier than 20 weeks of pregnancy and during the first trimester) [45,46], which facilitate early informed decision-making, including the option of early pregnancy termination and thus, decrease the probability of having newborns with congenital anomalies and its associated complications and hospitalisation. Termination of pregnancy practices differ from country to another based on their own regulations. In recent years the NHS has applied changes on how and where termination of pregnancy services are delivered. Geographical location impacted women access to termination services in the UK, it may be difficult for women who live in remote areas [47]. Furthermore, geographical variation impacts the prevalence of congenital anomalies due to the variation in exposure to teratogens, age profiles of areas and also genetic composition of the local population [48]. Besides, it might be difficult for women who are in the late second trimester of pregnancy or who have complex pre-existing medical conditions or difficult social circumstances. The abortion rate for women aged 15–44 years in England and Wales has increased markedly since 2018, (17.4 per 1000 resident). This is the highest abortion rate recorded, exceeding the previous peak in 2007, (17.9 abortions per 1000 resident). However, in our study, we found an increase in the trend of admissions due to congenital anomalies, which reflects an ongoing issue [1]. Congenital anomalies are gaining major representation in infant morbidity and mortality [7,49]. Which was also representing in our study as it showed increased in the trend of hospital admission due to congenital anomalies.

Congenital anomalies have a major impact on the health system, as around 3.3 million children (under five years) die from birth defects, and the 3.2 million who survive may develop a disability later in life [50]. In low/middle-income countries (LMIC), the mortality due to congenital anomalies for children aged under five years is likely to be fourfold underestimated due to the lack of actual causes of death [50,51,52]. In the UK, between 2005 and 2009, from 24,539 live born congenital anomalies cases registered, 1141 infants died due to these anomalies, which is not a small number [52]. In 2015, in England, it was reported that the highest mortality rates related to congenital anomalies were related to congenital heart defect (51%), chromosomal anomalies (28%), and digestive system anomalies (27%) [48]. Globally, congenital anomalies are also associated with a 40% increase in the length of stay in hospital compared to individuals with no underlying diseases [53]. A range of interventions have been proposed to reduce the burden of congenital anomalies and providing updated epidemiological data on the incidence of hospital admissions can provide important information to help plan and implement policy in the UK and worldwide.

### Strengths and Weaknesses of the Study

To the best of our knowledge, this is the first study to explore trends in the rates of hospital admissions due to congenital anomalies in England and Wales without restricting the study to specific inclusion/exclusion criteria. However, due to the nature of the data (on the population level) provided by the two databases we were not able to investigate the risk factors that may impact hospital admission. Moreover, ecological studies cannot establish causality. Other limitations include a lack of information on sex at the age-group level, rural/urban residence, and ethnicity for congenital anomalies data. Besides, types of data provided by the databases restricted our ability to investigate the association between congenital malformations-related hospital admissions and maternal age. In patients with multiple congenital malformations the dataset is limited to one malformation only, even if the reason for admission is the treatment of more than one malformation.

## 5. Conclusions

This was a preliminary study that showed that the trend of hospitalisation due to congenital anomalies in the UK has increased over the study period. The most common hospital admissions causes were congenital malformations of the circulatory system, the musculoskeletal system, genital organs, and the digestive system. Males had a higher percentage of hospitalisation compared to females. Further studies to investigate the factors associated with higher hospitalisation rate among males are needed. Besides, the clear increase in the admission rate among females warrant further investigation. Moreover, independent of sex, further studies are needed to explore other risk factors for congenital anomalies.

## Figures and Tables

**Table 1:** Percentage of congenital malformations, deformations and chromosomal abnormalities hospital admission from total number of admissions per ICD code.

ICD Code	Description	Percentage from Total Number of Admissions
Q00–Q07	Congenital malformations of the nervous system	3.6%
Q10–Q18	Congenital malformations of eye, ear, face and neck	7.9%
Q20–Q28	Congenital malformations of the circulatory system	20.1%
Q30–Q34	Congenital malformations of the respiratory system	2.7%
Q35–Q37	Cleft lip and cleft palate	3.1%
Q38–Q45	Other congenital malformations of the digestive system	12.0%
Q50–Q56	Congenital malformations of genital organs	14.2%
Q60–Q64	Congenital malformations of the urinary system	5.4%
Q65–Q79	Congenital malformations and deformations of the musculoskeletal system	19.5%
Q80–Q89	Other congenital malformations	10.1%
Q90–Q99	Chromosomal abnormalities, not elsewhere classified	1.3%

ICD = International Statistical Classification of Diseases system.

**Table 2:** Percentage change in the hospital admission rates for congenital malformations, deformations and chromosomal abnormalities from 1999–2019 in England and Wales.

Diseases	Rate of Diseases in 1999 per 100,000 Persons (95% CI)	Rate of Diseases in 2019 per 100,000 Persons (95% CI)	Percentage Change from 1999–2019
Congenital malformations of the nervous system	5.86 (5.65–6.07)	9.18 (8.93–9.42)	56.6%
Congenital malformations of eye, ear, face and neck	21.05 (20.65–21.44)	12.27 (11.99–12.55)	−41.7%
Congenital malformations of the circulatory system	31.57 (31.09–32.06)	50.68 (50.11–51.25)	60.5%
Congenital malformations of the respiratory system	3.67 (3.51–3.84)	7.39 (7.17–7.60)	101.1%
Cleft lip and cleft palate	6.77 (6.54–6.99)	5.90 (5.70–6.10)	−12.8%
Other congenital malformations of the digestive system	21.05 (20.66–21.44)	27.78 (27.35–28.20)	32.0%
Congenital malformations of genital organs	31.28 (30.80–31.76)	28.52 (28.10–28.95)	−8.8%
Congenital malformations of the urinary system	11.36 (11.07–11.65)	11.34 (11.07–11.61)	−0.2%
Congenital malformations and deformations of the musculoskeletal system	40.34 (39.79–40.88)	36.98 (36.49–37.47)	−8.3%
Other congenital malformations	22.97 (22.56–23.39)	15.80 (15.48–16.12)	−31.2%
Chromosomal abnormalities, not elsewhere classified	2.82 (2.68–2.96)	2.71 (2.58–2.84)	−3.8%

## Data Availability

Hospital admissions and population data are publicly available as de-identified data. Therefore, it was considered an exempt category.

## References

[1] World Health Organization (2020). Congenital Anomalies. https://www.who.int/news-room/fact-sheets/detail/congenital-anomalies.

[2] Sadler T. (2015). Birth defects and prenatal diagnosis. Langman’s Medical Embryology.

[3] World Health Organization (2009). Birth Defects. Report by the Secretariat. Sixty-Third World Health Assembly..

[4] El Koumi M.A., Al Banna E.A., Lebda I. (2013). Pattern of congenital anomalies in newborn: A hospital-based study. Pediatr. Rep..

[5] Naghavi M., Wang H., Allen C., GBD 2015 Mortality and Causes of Death Collaborators (2016). Global, regional, and national life expectancy, all-cause mortality, and cause-specific mortality for 249 causes of death, 1980–2015: A systematic analysis for the Global Burden of Disease Study 2015. Lancet.

[6] Ndibazza J., Lule S., Nampijja M., Mpairwe H., Oduru G., Kiggundu M., Akello M., Muhangi L., Elliott A. (2011). A description of congenital anomalies among infants in Entebbe, Uganda. Birth Defects Res. Part A Clin. Mol. Teratol..

[7] Boyle B., Addor M.-C., Arriola L., Barisic I., Bianchi F., Csáky-Szunyogh M., Walle H.E.K.D., Dias C.M., Draper E., Gatt M. (2018). Estimating Global Burden of Disease due to congenital anomaly: An analysis of European data. Arch. Dis. Child. Fetal Neonatal Ed..

[8] Gillani S., Kazmi N.H.S., Najeeb S., Hussain S., Raza A. (2011). Frequencies of congenital anomalies among newborns admitted in nursery of Ayub Teaching Hospital Abbottabad, Pakistan. J. Ayub Med. Coll. Abbottabad.

[9] Prashar N., Gupta S., Thakur R., Sharma P., Sharma G. (2016). A study of incidence of congenital anomalies in newborn: A hospital based study. Int. J. Res. Med Sci..

[10] Singh K., Krishnamurthy K., Greaves C., Kandamaran L., Nielsen A.L., Kumar A. (2014). Major Congenital Malformations in Barbados: The Prevalence, the Pattern, and the Resulting Morbidity and Mortality. ISRN Obstet. Gynecol..

[11] Taye M., Afework M., Fantaye W., Diro E., Worku A. (2019). Congenital anomalies prevalence in Addis Ababa and the Amhara region, Ethiopia: A descriptive cross-sectional study. BMC Pediatr..

[12] Public Health England (2018). National Congenital Anomaly and Rare Disease Registration Service: Congenital Anomaly Statistics 2018. https://www.gov.uk/government/uploads/system/uploads/attachment_data/file/630736/Congenital_anomaly_statistics_2015.pdf.

[13] Hobbs C., Cleves M., Simmons C. (2002). Genetic epidemiology and congenital malformations: From the chromosome to the crib. Arch. Pediatr. Adolesc. Med..

[14] Guiller C., Dupas G., Pettengill M. (2007). Child with congenital abnormality: Bibliographical study about pediatric nursing publications. Acta. Paul. Enferm..

[15] Horovitz D.D., Llerena J.C., Mattos R.D. (2005). Birth defects and health strategies in Brazil: Sn overview. Cad. Saude Publica.

[16] Health and Social Care Information Centre (HSCIC) (2021). Hospital Episode Statistics. http://http//content.digital.nhs.uk/hes.

[17] NHS Wales Informatics Service (2021). Annual PEDW Data Tables. http://www.infoandstats.wales.nhs.uk/page.cfm?pid=41010&orgid=869.

[18] Naser A.Y., Wang Q., Wong L.Y.L., Ilomaki J., Bell J.S., Fang G., Wong I.C.K., Wei L. (2018). Hospital Admissions due to Dysglycaemia and Prescriptions of Antidiabetic Medications in England and Wales: An Ecological Study. Diabetes Ther..

[19] Tulloch J.S.P., Decraene V., Christley R.M., Radford A.D., Warner J.C., Vivancos R. (2019). Characteristics and patient pathways of Lyme disease patients: A retrospective analysis of hospital episode data in England and Wales (1998–2015). BMC Public Health.

[20] Hemmo S.I., Naser A.Y., Alwafi H., Mansour M.M., Alanazi A.F.R., Jalal Z., Alsairafi Z.K., Paudyal V., Alomari E., Al-Momani H. (2021). Hospital Admissions Due to Ischemic Heart Diseases and Prescriptions of Cardiovascular Diseases Medications in England and Wales in the Past Two Decades. Int. J. Environ. Res. Public Health.

[21] Naser A.Y., Alrawashdeh H.M., Alwafi H., AbuAlhommos A.K., Jalal Z., Paudyal V., Alsairafi Z.K., Salawati E.M., Samannodi M., Sweiss K. (2021). Hospital Admission Trends due to Viral Infections Characterised by Skin and Mucous Membrane Lesions in the Past Two Decades in England and Wales: An Ecological Study. Int. J. Environ. Res. Public Health.

[22] NHS Wales Informatics Service (2021). Data Quality Status Report: Admitted Patient Care Data Set. http://www.infoandstats.wales.nhs.uk/documents/869/20191007-APCDQStatusReport2018-19-v1.pdf.

[23] Office for National Statistics (ONS) (2021). Population Estimates. https://www.ons.gov.uk/peoplepopulationandcommunity/populationandmigration/populationestimates/bulletins/annualmidyearpopulationestimates/mid2012tomid2016.

[24] Public Health England (2020). NCARDRS Statistics 2018: Summary Report. https://www.gov.uk/government/publications/ncardrs-congenital-anomaly-annual-data/ncardrs-statistics-2018-summary-report.

[25] Siddhisena D., Goel H. (2018). Congenital Anomalies Presenting to a Tertiary Neonatal Intensive Care Unit: A Descriptive Study. J. Birth Defects.

[26] Department of Health and Social Care (2020). Abortion Statistics, England and Wales: 2019.

[27] Bouma B.J., Mulder B.J.M. (2017). Changing Landscape of Congenital Heart Disease. Circ. Res..

[28] Hamamy H. (2012). Consanguineous marriages: Preconception consultation in primary health care settings. J. Community Genet..

[29] National Institute for Health and Care Excellence (2014). Risk Factors for Congenital Abnormalities. Eyes on Evidence..

[30] Goetzinger K., Shanks A., Odibo A., Macones G., Cahill A. (2017). Advanced Maternal Age and the Risk of Major Congenital Anomalies. Am. J. Perinatol..

[31] Ramakrishnan A., Lee L.J., Mitchell L.E., Agopian A.J. (2015). Maternal Hypertension during Pregnancy and the Risk of Congenital Heart Defects in Offspring: A Systematic Review and Meta-analysis. Pediatr. Cardiol..

[32] Etemad L., Moshiri M., Moallem S.A. (2012). Epilepsy drugs and effects on fetal development: Potential mechanisms. J. Res. Med. Sci..

[33] Shawky R., Sadik D.I. (2011). Congenital malformations prevalent among Egyptian children and associated risk factors. Egypt. J. Med. Hum. Genet..

[34] Abdou M.S.M., Sherif A.A.R., Wahdan I.M.H., Ashour K.S.E.D. (2019). Pattern and risk factors of congenital anomalies in a pediatric university hospital, Alexandria, Egypt. J. Egypt. Public Health Assoc..

[35] Dolk H., Loane M., Garne E. (2010). The Prevalence of Congenital Anomalies in Europe. Adv. Exp. Med. Biol..

[36] Dastgiri S., Stone D.H., Le-Ha C., Gilmour W.H. (2002). Prevalence and secular trend of congenital anomalies in Glasgow, UK. Arch. Dis. Child..

[37] Ajao A., Adeoye I. (2019). Prevalence, risk factors and outcome of congenital anomalies among neonatal admissions in OGBOMOSO, Nigeria. BMC Pediatr..

[38] Tennant P.W., Samarasekera S.D., Pless-Mulloli T., Rankin J. (2011). Sex differences in the prevalence of congenital anomalies: A population-based study. Birth Defects Res. Part A Clin. Mol. Teratol..

[39] Sokal R., Tata L.J., Fleming K.M. (2014). Sex prevalence of major congenital anomalies in the United Kingdom: A national population-based study and international comparison meta-analysis. Birth Defects Res. Part A Clin. Mol. Teratol..

[40] Elghanmi A., Razine R., Berrada R. (2017). Gender Difference in Specific Congenital Anomalies. World J. Res. Rev..

[41] Royal College of Obstetricians and Gynaecologists (2010). Termination of Pregnancy for Fetal Abnormality in England, Scotland and Wales. https://www.rcog.org.uk/en/guidelines-research-services/guidelines/termination-of-pregnancy-for-fetal-abnormality-in-england-scotland-and-wales/.

[42] National Health Service (2021). 20-Week Screening Scan. https://www.nhs.uk/pregnancy/your-pregnancy-care/20-week-scan/.

[43] Johnson S.P., Sebire N., Snijders R., Tunkel S., Nicolaides K. (1997). Ultrasound screening for anencephaly at 10-14 weeks of gestation. Ultrasound Obstet. Gynecol..

[44] Chitty L.S., Pandya P.P. (1997). Ultrasound screening for fetal abnormalities in the first trimester. Prenat. Diagn..

[45] AbdulAzeez S., Al Qahtani N.H., Almandil N., Al-Amodi A.M., Aldakeel S.A., Ghanem N.Z., Alkuroud D.N., Alturki A., Alqattan Q.A., Alghamdi A. (2019). Genetic disorder prenatal diagnosis and pregnancy termination practices among high consanguinity population, Saudi Arabia. Sci. Rep..

[46] Hamilton M.J., Eason J. (2015). Prenatal diagnosis of genetic disorders. Obstet. Gynaecol. Reprod. Med..

[47] National Institute for Health and Care Excellence (2019). Abortion Care. https://www.nice.org.uk/guidance/ng140/chapter/Recommendations.

[48] Public Health England (2017). National Congenital Anomaly and Rare Disease Registration Service. Congenital Anomaly Statistics 2015.

[49] Chung C.S., Myrianthopoulos N.C., Opitz J.M., Reynolds J.F. (1987). Congenital anomalies: Mortality and morbidity, burden and classification. Am. J. Med. Genet..

[50] Carmona R.H. (2005). The global challenges of birth defects and disabilities. Lancet.

[51] Sitkin N.A., Ozgediz D., Donkor P., Farmer D.L. (2015). Congenital Anomalies in Low- and Middle-Income Countries: The Unborn Child of Global Surgery. World J. Surg..

[52] Modell B., Berry R., Boyle C., Christianson A., Darlison M., Dolk H., Howson C.P., Mastroiacovo P., Mossey P., Rankin J. (2012). Global regional and national causes of child mortality. Lancet.

[53] Moorthie S., Blencowe H., Darlison M.W., Lawn J.E., Mastroiacovo P., Morris J.K., Modell B., Bittles A.H., Congenital Disorders Expert Group (2017). An overview of concepts and approaches used in estimating the burden of congenital disorders globally. J. Community Genet..

